# Identification of Novel Autoantibodies for Detection of Malignant Mesothelioma

**DOI:** 10.1371/journal.pone.0072458

**Published:** 2013-08-19

**Authors:** Xufei Zhang, Weike Shen, Xiaomin Dong, Jiangping Fan, Lixia Liu, Xu Gao, Kemp H. Kernstine, Li Zhong

**Affiliations:** 1 Department of Cell Biology, Hebei University College of Life Sciences, Baoding, Hebei, P.R.China; 2 University of Cincinnati College of Medicine, Cincinnati, Ohio, United States of America; 3 Division of Thoracic Surgery, UT Southwestern Medical Center, Dallas, Texas, United States of America; 4 Department of Basic Medical Sciences, Western University of Health Sciences, Pomona, California, United States of America; King's College London, United Kingdom

## Abstract

**Background:**

The malignant mesothelioma (MM) survival rate has been hampered by the lack of efficient and accurate early detection methods. The immune system may detect the early changes of tumor progression by responding with tumor-associated autoantibody production. Hence, in this study, we translated the humoral immune response to cancer proteins into a potential blood test for MM.

**Methodology/Principal Findings:**

A T7 phage MM cDNA library was constructed using MM tumor tissues and biopanned for tumor-associated antigens (TAAs) using pooled MM patient and normal serum samples. About 1008 individual phage TAA clones from the biopanned library were subjected to protein microarray construction and tested with 53 MM and 52 control serum samples as a training group. Nine candidate autoantibody markers were selected from the training group using Tclass system and logistic regression statistical analysis, which achieved 94.3% sensitivity and 90.4% specificity with an AUC value of 0.89 in receiver operating characteristic analysis. The classifier was further evaluated with 50 patient and 50 normal serum samples as an independent blind validation, and the sensitivity of 86.0% and the specificity of 86.0% were obtained with an AUC of 0.82. Sequencing and BLASTN analysis of the classifier revealed that five of these nine candidate markers were found to have strong homology to cancer related proteins (PDIA6, MEG3, SDCCAG3, IGHG3, IGHG1).

**Conclusions/Significance:**

Our results indicated that using a panel of 9 autoantibody markers presented a promising accuracy for MM detection. Although the results need further validation in high-risk groups, they provided the potentials in developing a serum-based assay for MM diagnosis.

## Introduction

Malignant mesothelioma (MM) is an extremely aggressive cancer that originates from mesothelial cells of the pleural membranes and peritoneal tissues [1]–[7]. Once considered rare, MM is increasing, with a peak in incidence predicted to occur between 2010 and 2025 [8]–[11]. Although the disease is not frequent, it is quite devastating, with a median survival of 7 months [12]–[16]. Since the onset of the disease is delayed for as much as 50 years beyond exposure of asbestos, symptoms are vague, and diagnostic tools are not sensitive and specific enough to detect the disease until it reaches advanced stages [17]. Therefore, novel strategies of MM early detection and screening are urgently needed for improving MM management.

Because diagnosis of MM requires distinguishing it from benign pleural disease or from metastasis of other primary cancers to the pleura[15], the current invasive detection procedures, such as pleural fluid cytology obtained through thoracentesis, needle biopsy of pleural tissue under CT guidance, and open thoracotomy have low sensitivity ranging from 0% with a single sampling to 64% with serial samplings [6], [18], [19]. Developing an accurate and non-invasive cancer screening test using molecular biomarkers has proven to be a very attractive but difficult task. A variety of MM tumor markers have been identified. Most are circulating proteins/antigens, either secreted or breakdown products of malignant cells, which can be measured clinically by immunoassay [8], [20]. Soluble mesothelin-related protein (SMRP), megakaryocyte potentiating factor (MPF), and mesothelin (MSLN) variants are the most commonly used serological tumor antigens for MM detecting. Measurement of SMRP levels is currently available in clinic, but 50% sensitivity and 72% specificity remain less impressive [8], [20]–[22]. Studies of MPF and MSLN demonstrated sensitivities and specificities of 74.2% and 90.4%, and 59.3% and 86.2%, respectively [23]. Although the specificity of these markers is high, the sensitivity is still unacceptable as a screening test for MM.

In contrast to testing of circulating tumor-associated antigens (TAAs) as biomarkers, the use of a panel of serum antibodies against TAAs may provide reliable information for cancer diagnosis and prognosis [24]–[27]. This approach takes the advantage of immunesurveillance, the capacity of the immune system to identify tumor-specific proteins and respond with corresponding autoantibodies [28]. MM is a pulmonary malignancy that appears to be immunogenic, based on a large number of studies in both animals and humans [29]–[31]. Clinical trials of various immunotherapeutic regimens in patients with MM have shown certain capacity to ameliorate the disease [29]. In addition, the growth of transplantable syngeneic murine MM cell lines, which induce a disease pathologically identical to the human condition [30], can be regulated by immunologic processes [31].

In this study, we interrogated a T7 MM phage library using MM patient and control serum samples to identify immunogenic phage-expressed proteins. Protein microarray and bioinformatics tools were used to select and profile a panel of autoantibody biomarkers for MM diagnosis.

## Materials and Methods

### Human Subjects

All specimens in this study including 5 MM tissue samples and 215 serum samples (108 MM patients and 107 normal controls) were obtained from the National Mesothelioma Virtual Bank under the approval of the Institutional Review Board (IRB) of University of Pennsylvania Medical Center [32]. All the samples were collected from individuals with histologically confirmed MM after written consent forms were obtained and the details were shown in Table 1.

**Table 1:** Clinical information for the training and validation samples.

Variable	Training set	Validation set
Patients with clinical information		
No. of Patients	53	50
Age (yr)	53–85	51–87
Gender	Female (n = 12)	Female (n = 10)
	Male (n = 41)	Male (n = 40)
Tumor Stage	Stage III (n = 21)	Stage III (n = 19)
	Stage IV (n = 32)	Stage IV (n = 31)
Normal controls with no cancer history		
No. of Samples	52	50
Age (yr)	53–85	51–87
Gender	Female (n = 11)	Female (n = 10)
	Male (n = 41)	Male (n = 40)

### Phage Library Construction and Biopanning

A T7-phage mesothelioma cDNA library was constructed using 2.54 g tissues from 5 MM patients. Total RNA was extracted and purified using RNeasy Mini Kit (Qiagen, Valencia, CA, USA). Poly (A) RNA was isolated from total RNA by Oligotex Direct mRNA Mini Kit (Qiagen). OrientExpress cDNA synthesis and cloning systems (Novagen, Billerrica, MA, USA) was used for the MM T7 phage cDNA library construction. In order to control the average insert size by adjusting the ratio of primers to sample RNA, random primers were used to synthesize cDNA during construction. After vector ligation and T7 packaging, cDNA phage library was constructed and the library titer was determined by plaque assay.

The constructed cDNA library was then biopanned with pooled sera from 5 MM patients and 5 normal controls to enrich for tumor-associated proteins as described in Zhong 2005 [33]. Briefly, to remove non-tumor specific proteins, the phage-display library was affinity selected by incubating with protein G agarose beads coated with antibodies from pooled normal sera (10 µl of normal serum, diluted 1:10). Unbound phages were separated from phages bound to antibodies in normal sera by centrifugation. The retrieved supernatant containing unbound phages was then biopanned against protein G-agarose beads coated with pooled patient sera and isolated from unbound phages by centrifugation. The bound phages were eluted with 1% sodium dodecyl sulfate (SDS) and centrifuged at 4°C. The eluents from each biopan was titered by plaque assay.

### High-throughput Microarray Screening

After 4 cycle of biopanning, 1008 individual phage clones were picked and inoculated into 96-well plates containing 200 µl BLT5403 culture medium (OD600  =  0.6) in each well. Liquid LB and empty T7 phages were used as negative controls in the same process. These 96-well plates were incubated at 37°C for 3 hours and then moved to room temperature overnight until the phages were completely lysed. The phage lysates were then robotically spotted in 4 replicates on Nexterion nitrocellulose slides (SCHOTT Nexterion, Mainz, Germany) using OmniGrid 100 Arrayer (GeneMachines,San Carlos, CA, USA).

**Figure 1:**
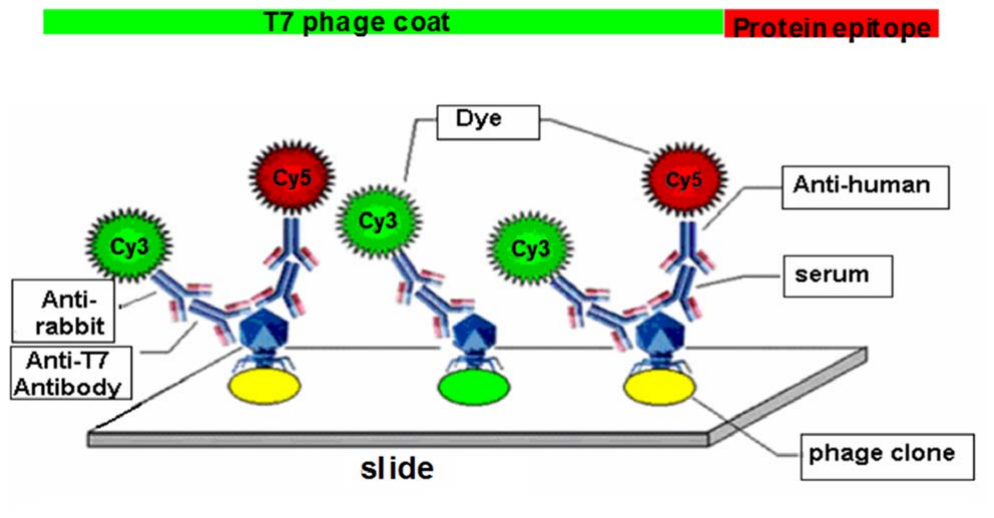
Dual-color fluorescent protein microarray detecting system. Sera from MM patients and normal donors were used as the source of primary antibodies for detecting phage-expressed, immunogenic proteins. Mouse anti-T7 antibody was used to detect the T7 phage capsid proteins as an internal control. Fluorescently labeled Cy5 anti-human and Cy3 anti-mouse secondary antibodies were used to visualize the primary antibodies. Each spot on the slide had a green signal from the T7 phage capsids, while the immunogenic phage clones had red signals from serum autoantibodies.

Two-color fluorescent detection was used to screen for immunogenic phage-displayed proteins (Fig. 1). Patient or control serum samples independent from biopanning were utilized as the primary antibody to detect the recombinant proteins, while the T7-tag monoclonal antibody was used to detect T7 capsid proteins as an internal control. Serum samples (1∶500) and T7-tag antibodies (1∶3000) were diluted with blocking buffer (1×PBS with 0.1% Tween-20 plus 3% skimmed milk) and tested with the microarray slides for 1 hour at room temperature. Slides were washed and then detected with Cy5-conjugated goat anti-human (diluted 1:500 in blocking buffer) and Cy3-conjugated goat anti-mouse (diluted 1∶3000 in blocking buffer) secondary antibodies (Jackson Immuno-Research, West Grove, PA, USA) for 1 hour in the dark at room temperature. Finally, Slides were washed three times in PBS with 0.1% Tween-20, and scanned using GenePix 4000B scanner (Molecular Devices, Sunnyvale, CA, USA). Each serum sample was repeated three times.

### Statistical Data Analysis

Microarray slides were scanned using 635 and 532 nm lasers which produce a red (Cy5) and green (Cy3) signals that were analyzed using GenePix 6.0 software. The median signal ratio of Cy5 and Cy3 were normalized using lowess smoothing in Matlab program to eliminate intensity-dependent variations [34]–[36]. Measurements were further normalized by subtracting background reactivity of serum against empty T7 phage proteins and dividing by the median of T7 signal [(Cy5:Cy3 of phage- Cy5:Cy3 of T7)/Cy5:Cy3 of T7] in each individual slide.

In this study, we adapted a novel classification method, Tclass, which was developed by Li et.al [37]–[40] as a method for statistical model. Briefly, the Tclass system combines Naive Bayes method and feature forward selection based on a stepwise optimization process for disease classification, in addition leave-one-out cross validation (LOOCV) was also incorporated into the system to evaluate classification accuracy. In this study, Tclass system automatically found the optimal combination of markers with the number of markers from 1 to 30. For each optimal combination, the 105 samples in the training group were randomly divided into two subgroups with partition ratio of 85%. The major subgroup was used to construct the classifier, and the minor subgroup was used to calculate classification accuracy by LOOCV. The above processes were repeated 1008 times and average classification accuracy was taken as stability index.

The classifiers were further examined by cluster heat maps and independent Student’s t-test. The coefficient of variations (CVs) among three replicate slides of each serum were calculated for selected candidate markers to measure the reproducibility of our microarrays. Logistic regression and receiver operating characteristic (ROC) analysis were used to evaluate the sensitivity and specificity for predictive accuracy. Statistical data analysis was performed using the Matlab, Cluster and Treeview software.

### Sequencing Identification

Phage identities were made based on significant nucleotide sequencing. The cDNA inserts were amplified by PCR using the T7 primers provided by the manufacturer and sequenced. The sequencing results were identified in the GenBank database using BLASTN. Uniprot and Wikigene search engines were used to get the proteins information in detail.

## Results

### Biopanning Enrichment of the Phage Library

A T7 MM phage cDNA library was constructed using pooled MM tissues. The quality of this library was titered by plaque assay and found to contain 5.6×10^6^ primary recombinants. The diversity of cDNA recombinants of the library was further examined by PCR amplification. The result from 100 randomly selected phage clones showed that 95% clones contained unique cDNA fragment inserts and sizes of the inserts ranged from 100 bp to 500 bp in length. In order to screen out the disease associated phage-displayed proteins, the phage library was biopanned using autoantibodies from pooled patient and pooled control serum samples. To determine the optimal cycles of biopanning, each cycle of biopanning was titered by plaque assay to calculate the remaining cDNA recombinants. The phage titers for each cycle of biopanning were BP1, 8.2×10^9 ^pfu/mL; BP2, 3.6×10^6 ^pfu/mL; BP3, 2.1×10^3 ^pfu/mL; and BP4, 1.3×10^3 ^pfu/mL. Since the biopanned library was not amplified during each cycle, the library titers appeared to reach a plateau between BP3 and BP4, which indicated the remaining library contained true specific disease-associated proteins. Therefore, we selected the output of BP4 as the candidate phage proteins for the protein microarray construction.

### Microarray Data Processing

**Figure 2:**
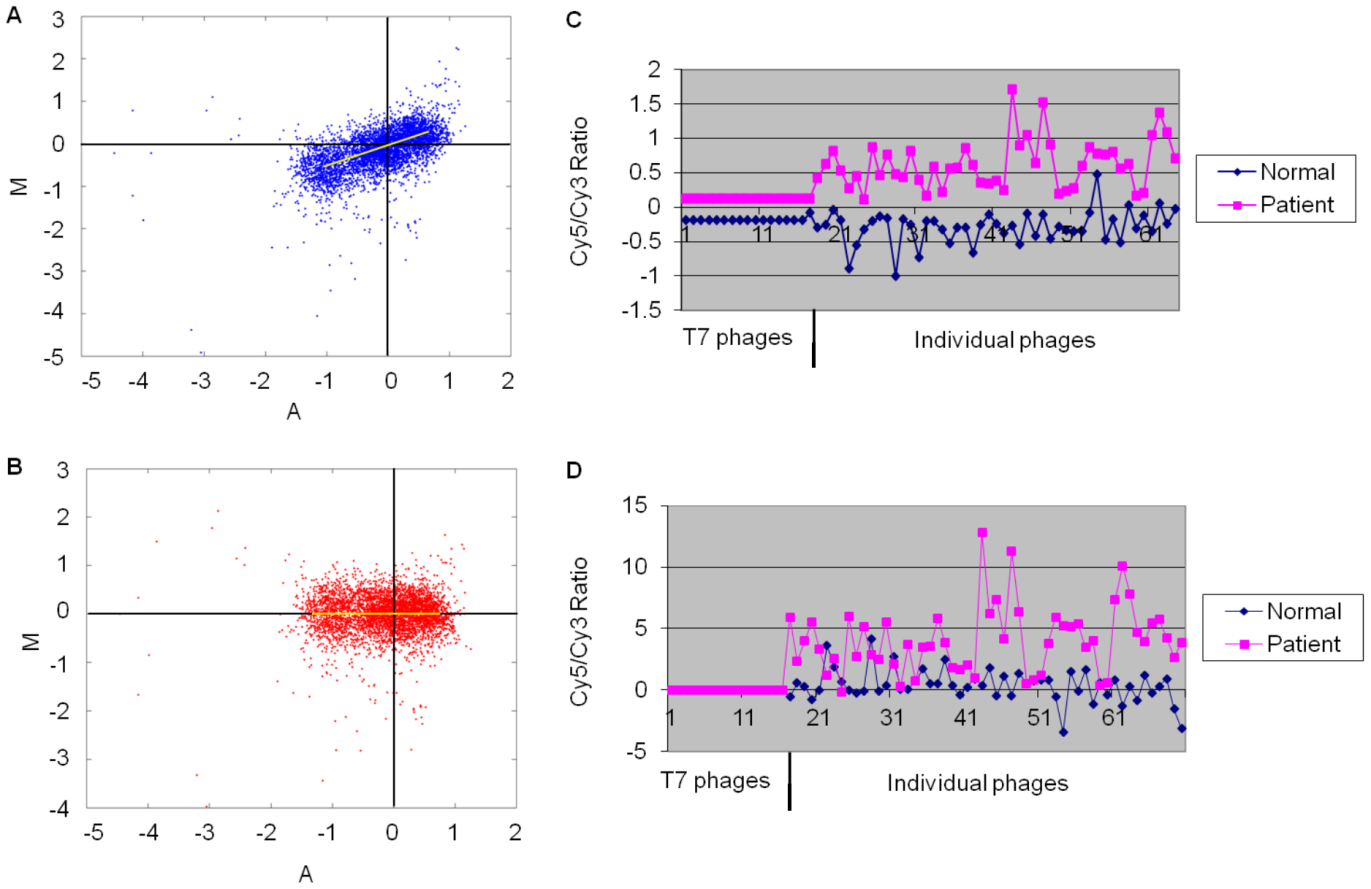
Lowess smoothing and T7 normalization. Lowess smoothing was used to remove intensity-dependent variations during fluorescence staining, A Lowess curve is calculated by fitting a line to the local neighborhood of each data point, and aggregating the line segments into a curve which is used to adjust each spot's value. A Lowess curve calculated after normalization should be a straight line with zero slope, indicating that ratio values are no longer dependent on intensity. (**A**) MA plot* before lowess smoothing, the majority of intensities were fluctuate. (**B**) MA plot after lowess smoothing, the majority of the point’s intensities were evenly distributed around zero. (**C**) Chip-to-chip variability was normalized by empty T7 phage proteins which were spotted as standard control on the slides. (**D**) Patterns of signal ratio distribution after normalization by empty T7 phages. *MA plot is a plot of the distribution of the red/green intensity ratio ('M') plotted by the average intensity ('A'). The equations for M & A are M = log_2_(R/G) = log_2_(R)-log_2_(G), A = 1/2log_2_(RG) = 1/2(log_2_(R)+log_2_(G)).The majority of the points on the y axis (M) would be located at 0, since Log (1) is 0. If this is not the case, then a normalization method like lowess should be applied.

**Figure 3:**
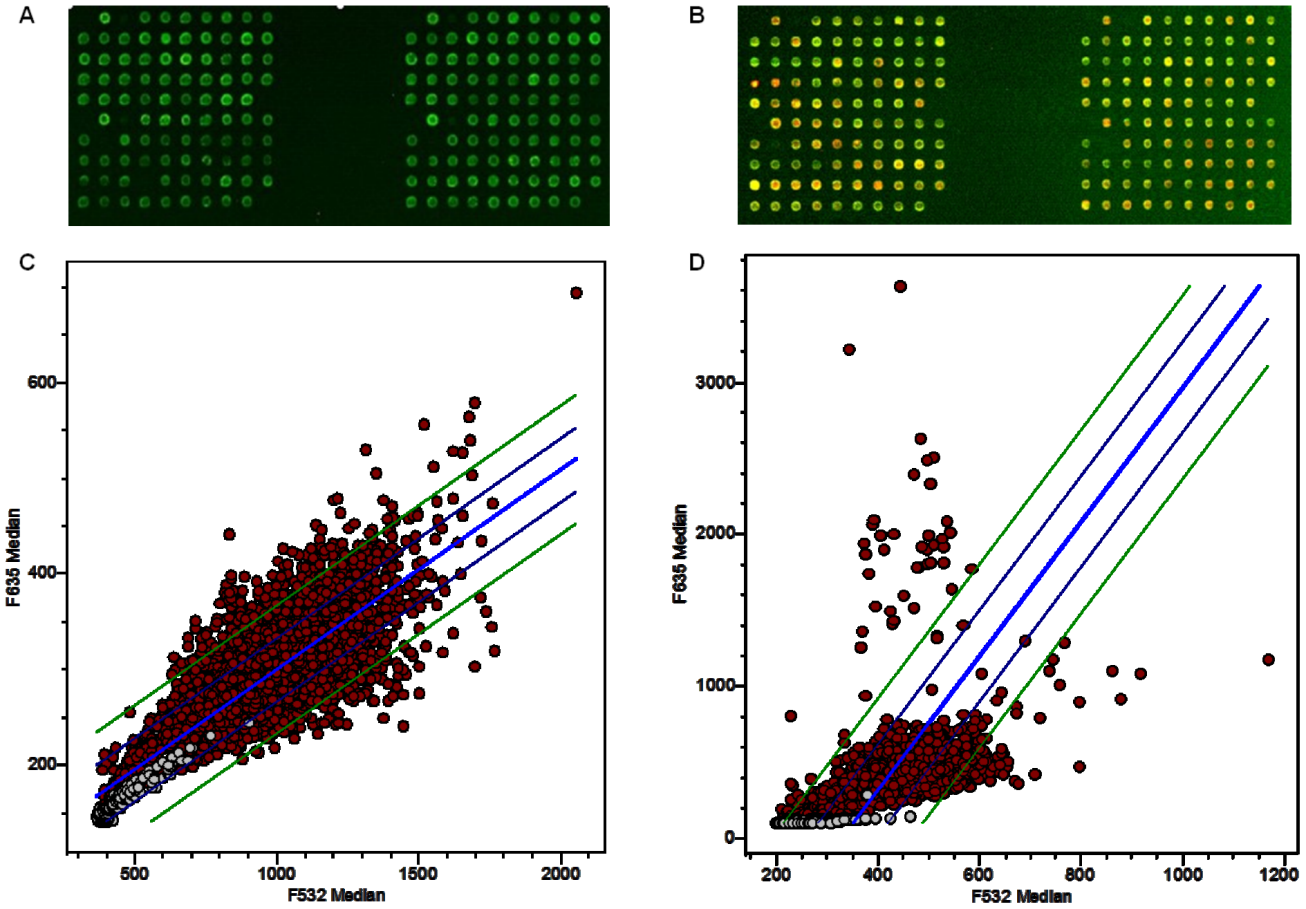
Protein microarray screening. Biopanned phage clones were spotted on microarray slides and tested with MM or normal serum samples. Scatter plots with linear regression show the differences between patient group and normal group. (**A**) The image of phage protein microarray tested by normal serum; (**B**) The same potion of the microarray tested by patient serum sample. (**C**) Linear regression was generated by GenePix software with normal sample. (**D**) Linear regression and 2SD lines were generated with patient sample.

A total of 1008 phage clones were randomly selected from the output of the BP4 phage library and spotted in 4 replicates onto nitrocellulose coated slides. To select the most representative clones for classifier development, 53 patient and 52 control serum samples that were not used in the biopanning were tested with the microarray slides as a training set. The intensity of Cy5 (red, signal of the recombinant protein) and Cy3 (green, signal of the T7 phage coat protein) of each clone was calculated in GenePix 6.0. In order to reduce the intensity-dependent variation in dye bias, lowess was used to apply a smoothing adjustment (Fig. 2). Chip-to-chip variability, a suspected variable of total IgG concentrations in individual serum samples, was normalized relative to the signal from empty T7 phage (Fig. 2). After normalization, Cy5/Cy3 ratio was calculated for each clone and linear regression of the Cy5/Cy3 signals on each slide was generated. By comparing the reactivity of patient and control samples, phage clones showed stronger immune-reactivity with patient sera were much more than with control sera (Fig. 3), indicating the effectiveness of the biopanning.

**Figure 4:**
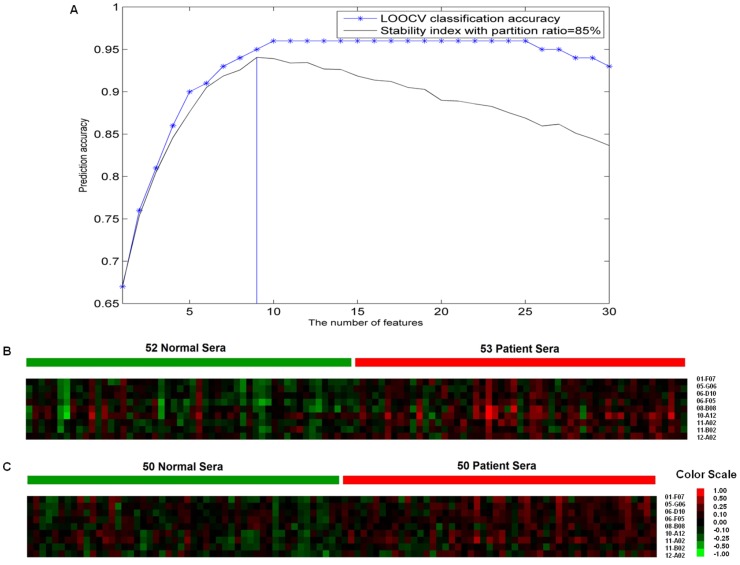
Markers classification and clustering. To classify the most optimal markers, the normalized data was analyzed using Naive Bayes method and evaluated by LOOCV in the Tclass system. The selected markers were analyzed using cluster heat map. (**A**) Tclass system automatically found the optimal combination of markers with the number of markers from 1 to 30. For each optimal combination, the 105 samples in the training group were randomly divided into two subgroups with partition ratio of 85%. The major subgroup was used to construct the classifier, and the minor subgroup was used to calculate classification accuracy by LOOCV. The above processes were repeated 1008 times and average classification accuracy was taken as stability index. Nine candidate markers were selected and then evaluated by LOOCV with a classifying accuracy of 95.0% and a stability of 94.1%. (**B**) Cluster heat map of 52 normal control samples and 53 MM patient samples in the training set. (**C**) Cluster heat map of 50 normal control samples and 50 patient samples in the validation group.

**Figure 5:**
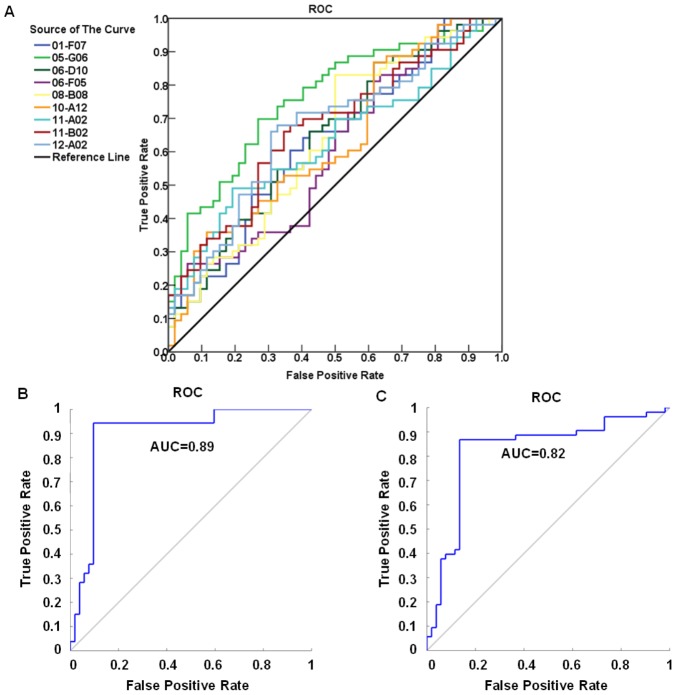
Classifier prediction. The classifier was evaluated by logistic regression with the individual and combined ROC. (**A**) Individual ROC for the training set. (**B**) Combined ROC curve for the training set, with an optimal sensitivity of 94.3% and specificity of 90.4%. (**C**) Combined ROC curve for the validation set, with sensitivity of 86.0% and specificity of 86.0%.

To select the most optimal markers, the normalized data was analyzed and evaluated using Naive Bayes classifier and LOOCV in the Tclass system. As a result, a panel of 9 candidate markers was selected by this classifier and then evaluated by LOOCV with a classifying accuracy of 95.0% and a stability of 94.1% (Fig. 4). The sensitivity of 94.3% and specificity of 90.4% for this panel were evaluated by logistic regression with an area under the ROC curve (AUC) value of 0.89 (Fig. 5). Individual ROC curves for these 9 candidate markers were shown in Fig. 5 as well. Sensitivity and specificity for individual candidate markers were shown in Table 2.

**Table 2:** Individual and combined performance of 9 candidate markers in training set.

Clone ID	AUC	Sensitivity	Specificity	P value	CVs*
01-F07	0.63	60.4%	63.5%	0.006	11%
05-G06	0.76	69.8%	73.1%	<0.0001	8%
06-D10	0.64	66.0%	57.7%	0.006	13%
06-F05	0.60	60.4%	51.9%	0.019	15%
08-B08	0.64	60.4%	57.7%	0.008	13%
10-A12	0.63	50.9%	67.3%	0.01	10%
11-A02	0.63	64.2%	51.9%	0.005	14%
11-B02	0.67	67.9%	63.5%	0.00058	11%
12-A02	0.67	71.7%	61.5%	0.003	12%
9-combined	0.89	94.3%	90.4%		

* The coefficient of variation (CV) is defined as the ratio of the standard deviation to the mean.

To further verify this panel, cluster heat maps were analyzed using Cluster & Treeview, which revealed the overview differences between patient and normal sera for this training group (Fig. 4). The independent student’s t-test result of most markers in this panel revealed statistically significantly different (P<0.01). The CVs of these 9 candidate markers ranged from 8% to 15% of mean among three replicate slides of each serum sample (Table 2).

### Validation of the Classifiers

After developing the classifier in the training set, this classifier was further cross-validated using an independent cohort of 50 MM patient and 50 control sera, which were not used previously. This cohort of samples was tested with the same protein microarray slides as in the training set. The corresponding data to the 9 markers was extracted and normalized as in the training set. The classifier was then used to predict the status of each sample in the validation set. The validated result showed that the sensitivity and specificity were 86.0% and 86.0%, respectively with AUC of 0.82 (Fig. 5).

### Characterization of the Panel Markers

The 9 phage proteins selected for classifier construction were sequenced and analyzed for their homologies to mRNA and genomic characters in GenBank database using BLASTN. Uniprot and Wikigene database were used to obtain the expressed protein identities. The searching results indicated that 5 of these 9 candidate markers were found to have known roles associated with cancer development, and the details were shown as follows: PDIA6 (protein disulfide isomerase family A, member 6)[41], [42], MEG3 (maternally expressed 3)[43], [44], SDCCAG3 (serologically defined colon cancer antigen 3)[45], [46], IGHG1 (Immunoglobulin heavy constant gamma 1)[47]–[49], IGHG3 (Immunoglobulin heavy constant gamma 3)[50]–[52], NADH dehydrogenase 1, BAC RP11-484D18, Clone CH507-528H12 on chromosome 21 and Clone RP11-413M3 on chromosome 9 (Table 3). The complete DNA sequences of these 9 phage inserts were provided as Supplementary Information (File S1).

**Table 3:** Blast results for 9 individual candidate markers.

Clone ID	Accession NO.	Details of homology status	Size (in bp)	Gene length (bp)	Position of tagged transcript	Max Score	Query Coverage%*	E Value*	Max Identity*
01-F07	NM_005742.2	PDIA6	272	2344	742–956	388	79%	1e-104	99%
05-G06	AF151783.1	MEG3	223	3768	1856–2011	289	69%	9e-75	100%
06-D10	AY349357.1	SDCCAG3	330	1134	568–840	505	82%	1e-139	100%
06-F05	AL592301.14	Clone RP11-413M3 on chromosome9	223	188462	89014–89119	196	47%	5e-47	100%
08-B08	FP236383.15	Clone 507-528H12 on chromosome 21	431	161389	87270–87633	669	84%	0.0	99%
10-A12	BC033178.1	IGHG3	359	1780	324–616	538	81%	1e-149	99%
11-A02	BC089417.1	IGHG1	335	1659	424–716	484	87%	2e-133	95%
11-B02	BC050745.1	NADH dehydrogenase1	274	2264	214–430	390	79%	3e-105	99%
12-A02	AC023235.24	BAC RP11-484D18	263	169633	121389–121533	268	55%	1e-68	100%

* Query coverage is percentage of the query length that is included in the aligned segments.

* E value is number of alignments that expected by chance with a particular score or better.

* Max identity refers to the alignment of the Blast input (query) sequence to its matched (subject) sequence and indicates the maximum percentage of identical nucleotides or amino acids within the noted alignment length.

## Discussion

Serological tumor markers have the potentials of being incorporated into diagnostic, prognostic and therapeutic practice in many cancers [53]–[57]. These goals have generated considerable interests in identifying predictive tumor markers over the past three decades [58], [59]. Many efforts had been focused on searching novel serological tumor-specific antigens in the past but with little success. In recent years, using tumor-associated autoantibodies as diagnostic biomarkers have been generating promising results for detection of breast cancer [57], head and neck cancer [60], prostate cancer [61] and lung cancer [33], [62]. There are several advantages of using serum autoantibodies as markers that make this approach more practical. First, non-invasive blood test makes it acceptable for most asymptomatic people. Second, antibodies are stable, with resistance to degradation, and are highly specific. Third, this is an efficient and low-cost method to detect cancer, allowing widespread implementation in resource-poor population [63].

In this study, we used phage display technology in combination with protein microarray for high-throughput quantitative analysis of potential autoantibody tumor markers using MM patient and control serum samples. Using Tclass system, we mined through a massive data set to classify a panel of 9 candidate markers. Five of these markers were found to represent or mimic known cancer antigens. LOOCV and logistic regression were further applied to validate the ability of this panel of markers for MM detection. Although the individual ROC and sensitivity and specificity of each marker were less impressive, combination of the 9 markers demonstrated significant increase in the diagnostic accuracy with 94.3% sensitivity and 90.4% specificity in the training set, and 86.0% sensitivity and 86.0% specificity in the validation set. The result indicated that there were great complementary among each markers in the classifier.

Like gene-expression profiling and other pattern-recognition approaches, protein microarray may also have the limitations of two-channel staining bias and chip-to-chip variability [61]. To minimize these problems, lowess smoothing and empty T7 phage normalization were introduced to reduce the variations in staining between chips (Fig. 4). In addition, in order to increase the selection of disease associated antigens in biopanned library, the output of the biopanned library was not amplified during each biopanning cycle. We have learned from our previous experiments that amplifying the output after each cycle of biopanning may increase in the number of redundant clones, and also decrease in getting more variety of real disease associated proteins, since amplifying could dilute the output of each biopanning. As a result, the titers of BP3 and BP4 showed a plateau which indicated true concentrated disease associated phage proteins were panned out. With a titer of 1.3×10^3 ^pfu/mL, we were nearly able to harvest all the phage clones after the BP4 by picking 1008 phage proteins for our protein microarray construction.

The tumor-associated proteins identified in study, PDIA6, MEG3, SDCCAG3, IGHG1 and IGHG3 have been previously reported to have cancer-associated properties. PDIA6 serves as regulators of both cell metabolism and stress response, and overexpression of PDLA6 is involved in different cellular processes, including cell migration and cell division in squamous cell carcinomas [41], [42]. MEG3 was reported to be a novel growth suppressor in human cancer that may play an important role in the development of human pituitary adenomas and bladder cancer cells [43], [44]. Recent studies demonstrated that SDCCAG3 expression level is elevated in colon cancers and SDCCAG3 is important for protein trafficking and for presentation of TNF receptor 1 on cell surface [45], [46]. IGHG1 is not exclusively to the immune cells, and it has been detected in human cancer tissue samples from breast, lung, and oral epithelial tumors, and also in human tumor cell lines. Additionally, the presence of IGHG1 is found in pancreatic cancer cells and might constitute an important element responsible for tumor cell proliferation and immune evasion mechanisms [47]–[49]. Several publications indicated that IGHG3 can be overexpressed in many different cancer cells and can differentiate tumor from normal [50]–[52]. The biological functions of IGHG1 and IGHG3 expression in cancer cells remains unclear despite some reports showing that IgG secreted by cancer cells had some unidentified capacity to promote the growth and survival of cancer cells. Although there was no study linking NADH dehydrogenase 1, BAC RP11-484D18, Clone CH507-528H12 on chromosome 21, and Clone RP11-413M3 on chromosome 9 to tumor development, the results of this study would provide useful information regarding their properties.

Although the result shown here appeared to be promising, this high accuracy in the laboratory may not hold true when increasing the sample size and extending our study to the early stage MM population. It would be our ultimate goal to develop such an assay that is able to detect MM from benign pleural disease and metastasis of other primary cancers to the pleura. So far, result in this manuscript is our first step towards developing such a clinic screening test. Since the disease is rare and the onset of the disease is delayed after asbestos exposure, it is difficult to get the early stage MM samples. Efforts are ongoing in recruiting serum samples from early stage MM patients as well as asbestos-exposed high-risk populations. Further validation will be carried out to evaluate the panel of autoantibodies identified in this study for the ability in detection of early stage disease. Ultimately, we are working towards the goal to develop a blood screening test for detection of MM disease in the high-risk populations.

Importantly, we have not exhaustively screened the phage library for all possible markers, and have likely not yet identified some significantly predictive circulating tumor-associated antibodies. It still needs more work to search for possible markers in this library and more data to validate the property and stability of this marker panel if it could be for clinical use. Further validation is needed using a large number of clinical samples in order to develop a screening test for early stage MM. These 9 novel disease markers may also have the potentials for prognostic and therapeutic usage.

## Conclusion

We identified a panel of 9 autoantibody markers that can provide encouraging accuracy for MM detection. Although the results need further validation in high-risk groups, they provided the potential to develop a serum-based assay for MM diagnosis.

## Supporting Information

File S1 **Individual DNA sequences of phage inserts for 9 candidate markers.**
(DOCX)
