# Supplementary material for: Identification of Novel Autoantibodies for Detection of Malignant Mesothelioma
Source: PLoS One. 2013 Aug 19;8(8):e72458. doi: 10.1371/journal.pone.0072458 (PMC3747111; doi:10.1371/journal.pone.0072458)
Supplement: File S1 — Individual DNA sequences of phage inserts for 9 candidate markers. (DOCX) [file pone.0072458.s001.docx]

01-F07

TCGAGTGCGGCCGCAAGCTTCAGAAGTAAAAGAGCAGACAGAAAGGAAAAGTGAAACTGGCAGCTGTGGATGCTACAGTCAATCAGGTTCTGGCCTCCCGATACGGGATTAGAGGATTTCCTACAATCAAGATATTTCAGAAAGGCGAGTCTCCTGTGGATTATGACGGTGGGCGGACAAGATCCGACATCGTGTCCCGGGCCCTTGATTTGTTTTCTGATAACGCTTGAATTCCGATCCCCGAGCATCACACCTGACTGGAATACAACAGC

05-G06

TCTCGAGTGCGGCCNCNNCTTGGGGCTCTCAGGCCGCAGACCTTGGGCCAGCAGGCCTCGGATCTCAGTGACACCGTCCGGGGACGCAGGTGGTGGTGACTCAGGGCTAGCCTCAAAGGGCAGCCCCACCTCCTCATCCTGGACCACAGAGACCACCTGCTTGGCGCGCCGTCGCTTGCTTGAATTCGGATCCCCGAGCATCACACCTGACTGGAATACGACA

06-D10

AGTTCTCGAGTGCGNCCGCAAGCTTCCCTAGCGCAGACTTTGCGGTTCATGGAGAGTCTCTGGGAGACAGGCACCTGCGGACGCTGCAGATAAGTTACGACGCACTGAAAGATGAAAATTCTAAGCTGAGAAGAAAGCTGAATGAGGTTCAGAGCTTCTCTGAAGCTCAAACAGAAATGGTGAGGACGCTTGAGCAGAAGTTAGAAGCAAAAATGATCAAGGAGGAAAGCGACTACCACGACCTGGAGTCGGTGGTTCAGCAGGTGGAGCAGAACCTGGAGCTGATGACCAAACGGGCTATCCCCGAGCATCACACNTGACTGGAATACG

06-F05

TTCTCGAGTGCGGCCGCAAGCTTCCCCGTGCTGTTGAAGGGAGAGGACCGGGACTGAGGCGACAACTCAAACCCCCTGCACCCCTCCTGGAGCTCCCAAACAGTTTTGTTAAAACAAGTGCAATTTTTTTCTTGGTGCCAGCAGCCGCGGTAATTCCAGCTCCAATAGCGTATATTAAAGTTGCTGCAGTTAAAAAGCTCGTANTTGGATCTTGGGAGCGGGC

08-B08

TCTCGAGTGCGGCCGCNNCTTCAGCTATCCTGAGGGAAACTTCGGAGGGAACCAGCTACTAGATGGTTCGATTAGTCTTTCGCCCCTATACCCAGGTCGGACGACCGATTTGCACGTCAGGACCGCTACGGACCTCCACCAGAGTTTCCTCTGGCTTCGCCCTGCCCAGGCATAGTTCACCATCTTTCGGGTCCTAACACGTGCGCTCGTGCTCCACCTCCCCGGCGCGGCGGGCGAGACGGGCCGGTGGTGCGCCCTCGGCGGACTGGAGAGGCCTCGGGATCCCACCTCGGCCGGCGAGCGCGCCGGCCTTCACCTTCATTGCGCCACGGCGGCTTTCGTGCGAGCCCCCGACTCNCGCACGTGTTAGACTCCTTGGTCCGTGGCTTGAATTCGGATCCCCGAGCATCACACCTGACTGGAATACGACA

10-A12

TCTCGAGTGCGGCCACTACTTAGATTCACCATCTTAAGAGATGATTCGAAGAACTCAGTGCATCTCCAAATGAACAGCCTCAAAACCGACGACACGGCCGTGTATTATTGTGTTAGAGATCTTGAGGGGGCTGGTAAATACGACTGGTATTTCGATATTTGGGGCCGAGGCATCCTGGTCACTGTCTCCTCAGCTNCCACCAAGGGCCCATCGGTCTTCCCCCTGGCGCCCTGCTCCAGGAGCACCTCTGGGGGCACAGCGGCCCTGGGCTGCCTGGTCAAGGACTACTTCCCCGAACCGGTGACGGTGTCGTGCTTGAATTCGGATCCCCGAGCATCACACCTGACTGGAATACGACA

11-A02

TCAGCGAGCCGGGTATAGTAGTGGCTGGAGGTGCTTTTGATATCTGGGGCCAAGGGACAATGGTCACCGTCTCTTCAGCCTCCACCAAGGGCCCATCGGTCTTCCCCCTGGCACCCTCCTCCAAGAGCACCTCTGGGGGCACAGCGGCCCTGGGCTGCCTGGTCAAGGACTACTTCCCCGAACCGGTGACGGTGTCNTGGAACTCANGCGCCCTGACCAGCGGCGTGCACACCTTCCCGGCTGTCCTACAGTCCTCAGGAGTCTANTCCCTCANNANCGTGNTGACNCTGCCCTCCANCAGCTTGGGCACCCNNACCTACATCTGNAAANGAATC

11-B02

AGTGCGGCCGCAAGCTTAGGGGCAGAAGCCAGAGGAAACTCTGGTGGAGGTCCGTAGCGGTCCTGACGTGCAAATCGGTCGTCCGACCTGGGTATAGGGGCGAAAGACTAATCAAACCATCTAGTAGCTGGTTCCCTCCGAAGTTTCCCTCAGGATAGCTGGCGCTCTCGCAGACCCGACGCACCCCCGCCACGCAGTTTTATCCGGTAAAGCGAATGATTAGAGGTCTTGGGGCCGCTTGAATTCGGATCCCCGANCATCNCACCTGCNCTGG

12-A02

TTACTCNAGTGCGGCCGCAAGCTTTGGGAGGCCAAGGCAGGCAGATCACCTGAGGTCAGGAGTTTGACACCAGCCTGATCAACATGGTGAAACCCCATCTCTACAAAAAAAAATACAAAAAAATTACCCAGGCATGGTGGCGCAGGCCTGTAATCTCAGCTACTCGACTATAGGCTTTCCTTCTCCATGATTCCCCAACTCTATACATCTTACGCTTGAATTCGGATCCCCGAGCATCACACCTGACTGGAATACGACAGCTC
